# Effect of Socioeconomic Status on Teeth and Dental Care – Evidence from a Population-based Study in Indonesia

**DOI:** 10.3290/j.ohpd.b3956549

**Published:** 2023-03-15

**Authors:** Ninuk Hariyani, Dini Setyowati, Stefan Listl, Rahul Nair

**Affiliations:** a Adjunct Researcher, Australian Research Centre for Population Oral Health, Adelaide Dental School, The University of Adelaide, Adelaide, South Australia, Australia; Senior Lecturer, Department of Dental Public Health, Faculty of Dental Medicine, Universitas Airlangga, Indonesia. Drafted the manuscript, data analysis, interpreted results, aquired funding, read and approved the final manuscript.; b Senior Lecturer, Department of Dental Public Health, Faculty of Dental Medicine, Universitas Airlangga, Indonesia. Critically revised the manuscript, acquired funding, read and approved the final manuscript.; c Professor, St Radboud University, Nijmegen, The Netherlands. Critically revised the manuscript, read and approved the final manuscript.; d Associate Professor, Australian Research Centre for Population Oral Health, Adelaide Dental School, The University of Adelaide, Adelaide, South Australia, Australia. Drafted the manuscript, data analysis, interpreted results, aquired funding, read and approved the final manuscript.

**Keywords:** edentulism, educational inequality, dental service utilisation, population-based study, toothbrushing frequency

## Abstract

**Purpose:**

Education is well-known as a determinant of oral health and dental behaviours in high-income countries, but much less is known for countries with lower incomes. This study aimed to identify the extent to which education affects oral health and dental behaviours in Indonesia.

**Materials and Methods:**

This study used data from the Indonesian Basic Health Survey 2013. From this nationally representative sample of 945,057 people 5–100 years old, a series of mixed-effects Poisson regression models that accounted for sampling weights estimated the effect of educational attainment on edentulism, dental care utilisation, and toothbrushing behaviour.

**Results:**

Consistent educational gradients were found for all outcomes and across all model specifications. People without a formal educational degree had a 1.03 (95% CI: 1.03–1.04) times higher risk of not utilising any dental care, a 3.15 (95% CI: 2.47–4.02) times higher risk of being edentulous, and a 15.6 (95% CI: 12.76–19.02) times higher risk of having low toothbrushing frequency than people having a university degree or higher.

**Conclusions:**

Stark and consistent educational gradients were observed in the dentate status, dental services utilisation, and toothbrushing in Indonesia. Educational inequalities were much larger for toothbrushing behaviours than for dental care utilisation. Intervention points for health policy should urgently prioritise public health interventions to promote overall educational attainment, preventive services, and dental care targeted at those with lower educational attainment.

The United Nation’s fourth sustainable development goal for 2030 aims to provide children across the planet with quality education.^24^ The largest gains for this ambitious goal will be made in less-developed nations.^18^ Education is emphasised as an important global goal, as greater educational attainment leads to greater economic and social opportunities.^24,25^ Due to the critical part it plays, educational attainment is an essential indicator of socioeconomic status (SES) later in life.^1,2^ Measures of SES locate individuals on the social and economic hierarchies in their community.^1,6^

The measurement of overall educational attainment is often a retrospective question, and its measurement is well understood.^21^ It is also relatively free of recall bias, although establishing cross-national equivalence has considerable limitations.^26^ In the context of health, it is well understood that educational attainment influences health. This makes the measurement of educational attainment in studies pertaining to healthcare vital, either as variables of interest or as variables that are adjusted for confounding and analysed to remove their biasing effects.^6^

Analysing the health status of individuals across various levels of educational attainment shows gradients on which less desirable health status is found more often among those with lower educational attainment. These gradients also exist between lower income/socioeconomic status and health. Similar findings were also found in different regions, including Chile, China, Finland, Australia, Scotland, and the United States. These findings and prior research, which show the interrelatedness of education and other indicators of SES, suggest similar trends among the various indicators of SES across geopolitical regions.

The population of Indonesia – the fourth highest in the world – is over 250 million, with more than 300 ethnicities spread across 6000 inhabited islands.^23^ The country has much lower literacy levels than other Southeast Asian nations.^3^ The percentage of Indonesians over the age of 25 who attained at least a bachelor’s degree in 2016 was just under 9%, the lowest of all the member states of the Association of Southeast Asian Nations (ASEAN). Health inequality in Indonesia was determined by a comprehensive assessment conducted by World Health Organization and the Indonesian Ministry of Health.^27^ It highlighted inequalities in over 50 health indicators across 11 health topics disaggregated by dimensions of inequality, such as household economic status, education level, place of residence, age, or sex. However, studies about dental health inequality in the Indonesian context are rare. Among the few, one study showed that those with low social capital were associated with edentulism.^17^ Previous research on the utilisation of dental care among Indonesians showed that dental utilisation depends on the ability to pay rather than the need for care. Inequality based on ability to pay persisted from 1999 to 2009.^15^ A previous study showed that lower socioeconomic status was associated with poor toothbrushing frequency.^19^

In the Indonesian context, where educational attainment continues to lag behind the rest of the region, its effect on oral health can illuminate an essential dimension of inequality that has notable recognition in the WHO’s Sustainable Development Goals (SDG). Thus, this study aimed to assess the differences in edentulism, dental care utilisation, and toothbrushing frequency based on educational attainment.

## Materials and Methods

The data used for this analysis are available upon written request to the Ministry of Health, Republic of Indonesia (http://labmandat.litbang.kemkes.go.id/images/download/peraturan/alur.pdf)

### Study Population and Research Design

Data from the 2013 Indonesian Basic Health Survey (Riskesdas 2013)^22^ were used for this study. Riskesdas 2013 is a cross-sectional national survey that is part of a serial Indonesian national basic health survey (http://labdata.litbang.kemkes.go.id/images/download/laporan/RKD/2013/Laporan_riskesdas_2013_final.pdf). It used a three-stage, stratified cluster sampling design to select a representative sample of Indonesian residents. The sampling frame was households recorded in the 2010 bloc census database, revalidated by the 2013 enumerator team. Indonesia was stratified into metropolitan and non-metropolitan areas by provincial status, with clusters based on district or municipality, which were selected with probability proportional to size. All persons in the household were included in the census. The final respondents were 294,959 households, with the mean number of residents per household equaling 3.8. The response rate for the Indonesian residents was 93%. Further details of the 2013 Indonesian national basic health research report have been published elsewhere.^22^

### Data Collection and Management

Data were collected via an interviewer-administered questionnaire. The outcomes of interest include dentate status, dental service utilisation, and toothbrushing frequency. Dental status was assessed through the self-reported question: ‘Have you lost all of your teeth?’, and answers were recorded as as dentate vs edentate. The respondents’ self-reported dental service utilisation was determined by answering the question, “Have you received dental treatment(s) during the last twelve months?”. The response options were yes or no. Toothbrushing frequency was determined by answering the question “Do you brush your teeth every day?”, with the response options being yes or no. Respondents with yes answers were categorised as having good toothbrushing frequency. Socioeconomic status was measured through education. Educational attainment was measured according to completion of various levels of schooling, post-school training, or tertiary educational attainment, and confounding variables were age, sex, and residential location. Age in years was used as a continuous variable. Sex was recorded as female or male. Residential location was dichotomised into urban or rural areas.

### Statistical Analysis

Statistical analyses were performed using STATA 15 (Stata; College Station, TX, USA). Descriptive analyses were carried out for the variables across the levels of educational attainment. Then, multivariable analyses that accounted for sampling weights were carried out for all analyses. Mixed-effects Poisson regression models that considered sampling weights were used with exponential estimates to report the relevant prevalence ratios. The three outcome variables, namely edentulism, dental services utilisation, and toothbrushing frequency, were modeled separately. The reference category for educational attainment was the highest level, which was a university degree or higher. The model for edentulousness included all participants. In contrast, toothbrushing frequency and dental services utilisation were recorded only among the dentate. The confounding variables (age, sex, and residential location-urban/rural) were entered into the models separately in blocks.

### Ethics Review

Ethical approval of Riskesdas 2013 was obtained from the Ministry of Health, Republic of Indonesia’s Human Research Ethics Committee. Since this study carried out secondary data analyses, new ethics clearance was not required.

## Results

945,057 people 5–100 years old responded. Of these, 770,422 individuals 12–100 years old were dentate. Table 1 shows the characteristics of study participants and the study characteristics based on the participants’ educational attainment. There were differences between the two populations in all characteristics presented, except the dental service utilisation pattern. In general, dentate respondents were better educated, slightly older people, women, urban residents, and people with good toothbrushing frequency.

**Table 1:** Characteristics of the study population based on educational attainment

Independent variables	Education						
	Never gained a formal education	Did not pass elementary school	Completed elementary school	Completed junior high school	Completed senior high school	Trade school or diploma	University degree or higher (ref)
	% and 95% CI	% and 95% CI	% and 95% CI	% and 95% CI	% and 95% CI	% and 95% CI	% and 95% CI
N (%) [95% CI]	79,270 (8.6 [8.5–8.6])	207,987 (22.5 [22.4–22.6])	256,887 (27.8 [27.7–27.9])	154,676 (16.7 [16.7–16.8])	175,349 (19.0 [18.9–19.0])	21,992 (2.4 [2.3–2.4])	28,579 (3.1 [3.1–3.1])
Age							
Min – max	6–100	6–100	10–100	12–98	15–98	16–98	19–97
(Mean [95% CI])	36.5 [36.3–36.7]	26.4 [26.3–26.5]	37.5 [37.4–37.6]	31.2 [31.1–31.3]	34.4 [34.4–34.5]	38.5 [38.3–38.7]	39.4 [39.2–39.5]
Sex							
Male	43.1 [42.7–43.4]	48.8 [48.6–49.0]	47.3 [47.2–47.5]	49.8 [49.5–50.0]	53.3 [53.0–53.5]	44.3 [43.7–45.0]	52.9 [52.3–53.5]
Female	56.9 [56.6–57.3]	51.2 [51.0–51.4]	52.7 [52.5–52.8]	50.2 [50.0–50.5]	46.7 [46.5–47.0]	55.7 [55.0–56.3]	47.1 [46.5–47.7]
Residential location							
Urban	31.9 [31.6–32.3]	37.3 [37.1–37.5]	37.4 [37.3–37.6]	47.7 [47.5–48.0]	63.2 [63.0–63.4]	68.3 [67.7–68.9]	74.8 [74.3–75.3]
Rural	68.1 [67.7–68.4]	62.7 [62.5–62.9]	62.6 [62.4–62.7]	52.3 [52.0–52.5]	37.0 [36.6–37.0]	31.7 [31.1–32.3]	25.2 [24.7–25.7]
Dental status							
Dentate	90.1 [89.9–90.4]	95.3 [95.2–95.4]	97.4 [97.4–97.5]	99.2 [99.1–99.2]	99.4 [99.3–99.4]	99.0 [98.9–99.2]	99.4 [99.3–99.5]
Edentulous	9.9 [9.6–10.1]	4.7 [4.6–4.8]	2.6 [2.5–2.6]	0.8[0.8–0.9]	0.6 [0.6–0.7]	1.0 [0.8–1.1]	0.6 [0.5-0.7]
Dental service utilisation in the last 12 months							
Yes	7.3 [7.1–7.4]	8.2 [8.1–8.3]	8.0 [7.9–8.1]	8.2 [8.0–8.3]	9.1 [9.0–9.3]	10.1 [9.7–10.5]	11.9 [11.5–12.3]
No	92.8 [92.6–92.9]	91.8 [91.7–91.9]	92.0 [91.9–92.1]	91.8 [91.7–92.0]	90.9 [90.7–91.0]	89.9 [89.5–90.3]	88.1 [87.7–88.5]
Toothbrushing frequency							
Good	65.2 [64.8–65.6]	85.2 [85.0–85.4]	91.1 [91.0–91.2]	95.8 [95.7–95.9]	97.2 [97.1–97.2]	97.3 [97.0–97.5]	98.4 [98.2–98.5]
Bad	34.8 [34.4–35.2]	14.8 [14.6–15.0]	8.93 [8.82–9.04]	4.24 [4.14–4.34]	2.84 [2.76-2.92]	2.75 [2.54–2.97]	1.62 [1.48–1.77]

95% CI: 95% confidence interval.

Table 2 presents a multivariable analysis of edentulism among all respondents. The adjusted prevalence ratios among people with less education decreased from a PR of 24.71 (95% CI = 19.41–31.45) to 3.15 (95% CI = 2.47–4.02) in the fully adjusted model. There was a clear gradient showing that people with less education were at a higher risk of being edentulous. In the fully adjusted model, people without a formal education and those who did not pass elementary school had a 3.15 (95% CI = 2.47–4.02) and a 3 (95% CI = 2.35–3.81) times higher risk of being edentulous than those with a university degree or higher, respectively. Similarly, those who completed (senior) high school or those who had completed a course at trade school or a diploma had a 1.45 (95% CI = 1.12–1.86) or 1.55 (95% CI = 1.13–2.12) times higher chance of edentulousness, respectively.

**Table 2:** Multivariable analysis of edentulism in Indonesia

	Unadjusted model	Adjusted for age and sex	Adjusted for age, sex and residential location
	PR [95% CI]	PR [95% CI]	PR [95% CI]
		N = 945,057	
Education			
Never gained a formal education	**24.71 [19.41–31.45]**	**3.47 [2.72–4.41]**	**3.15 [2.47–4.02]**
Did not pass elementary school	**9.89 [7.77–12.60]**	**3.26 [2.56–4.15]**	**3.00 [2.35–3.81]**
Completed elementary school	**4.84 [3.80–6.16]**	**2.81 [2.22–3.57]**	**2.62 [2.06–3.32]**
Completed junior high school	**1.42 [1.10–1.83]**	**2.05 [1.60–2.63]**	**1.98 [1.54–2.54]**
Completed senior high school	**1.06 [0.82–1.37]**	**1.47 [1.14–1.89]**	**1.45 [1.12–1.86]**
Trade school or diploma	**1.66 [ 1.20–2.30]**	**1.57 [1.14–2.15]**	**1.55 [1.13–2.12]**
University degree or higher (ref)	**–**	**–**	**–**
Age	**–**	**1.09 [1.05–1.14]**	**1.10 [1.06–1.14]**
Sex (ref male)	**–**	**1.09 [1.09–1.09]**	**1.09 [1.09–1.09]**
Residential location (ref urban)	**–**	**–**	**1.19 [1.14–1.24]**

PR: prevalence risk; 95% CI: 95% confidence interval. Boldface indicates statistical significance.

The multivariable analysis of dental service utilisation among dentate respondents is presented in Table 3. There was a decreasing likelihood of dental service utilisation with lower levels of education. Those with no formal education (PR = 1.03; 95% CI = 1.03–1.04) and those who had not completed elementary school (PR = 1.02; 95% CI = 1.02–1.03) were the least likely to visit the dentist.

**Table 3:** Multivariable analysis of dental service utilisation (no dental treatment in the last 12 months among dentate Indonesians)

	Unadjusted model	Adjusted with age and sex	Adjusted for age, sex and residential location
	PR [95% CI]	PR [95% CI]	PR [95% CI]
		N = 770,422	
Education			
Never gained a formal education	**1.03 [1.02–1.03]**	**1.03 [1.03–1.04]**	**1.03 [1.03–1.04]**
Did not pass elementary school	**1.02 [1.02–1.03]**	**1.02 [1.02–1.03]**	**1.02 [1.02–1.03]**
Completed elementary school	**1.02 [1.02–1.02]**	**1.02 [1.02–1.02]**	**1.02 [1.02–1.02]**
Completed junior highschool	**1.02 [1.02–1.02]**	**1.02 [1.02–1.02]**	**1.02 [1.02–1.02]**
Completed senior highschool	**1.02 [1.01–1.02]**	**1.01 [1.01–1.02]**	**1.01 [1.01–1.02]**
Trade school or diploma	**1.01 [1.01–1.01]**	**1.01 [1.01–1.01]**	**1.01 [1.01–1.01]**
University degree or higher (ref)	**-**		
Age	**-**	**0.99 [0.99–0.99]**	**0.99 [0.99–0.99]**
Sex (ref male)	-	1.00 [1.00–1.00]	1.00 [1.00–1.00]
Residential location (ref urban)	-	-	1.00 [1.00–1.00]

PR: prevalence risk; 95% CI: 95% confidence interval. Boldface indicates statistical significance.

Table 4 presents a multivariable analysis of poor toothbrushing frequency among dentate respondents. Compared to people with a university degree or higher, people with lower education have a higher risk of having poor toothbrushing frequency, ranging from a PR =1.52 and 95% CI = 1.17–1.96 to a PR = 15.58 and 95% CI = 12.76–19.02 among respondents with a trade-school education or a diploma degree to those with no formal educational background, respectively.

**Table 4:** Multivariable analysis of poor toothbrushing frequency among dentate Indonesians

	Unadjusted model	Adjusted with age and sex	Adjusted for age, sex and residential location
	PR [95% CI]	PR [95% CI]	PR [95% CI]
		N = 770,422	
Education			
Never gained a formal education	**31.35 [25.69–38.26]**	**21.75 [17.82–26.55]**	**15.58 [12.76–19.02]**
Did not pass elementary school	**12.58 [10.31–15.36]**	**11.24 [9.21–13.71]**	**8.39 [6.87–10.24]**
Completed elementary school	**6.14 [5.03–7.50]**	**6.21 [5.09–7.58]**	**4.75 [3.89–5.80]**
Completed junior high school	**3.01 [2.46–3.69]**	**3.77 [3.08–4.62]**	**3.16 [2.58–3.87]**
Completed senior high school	**1.95 [1.59–2.39]**	**2.25 [1.83–2.76]**	**2.11 [1.72–2.59]**
Trade school or diploma	**1.48 [1.14–1.91**	**1.58 [1.22–2.05]**	**1.52 [1.17–1.96]**
University degree or higher (ref)	**–**	**–**	**–**
Age	**–**	**0.62 [0.61–0.64]**	**0.63 [0.62–0.65]**
Sex (ref male)	**–**	**1.03 [1.03–1.03]**	**1.03 [1.03–1.03]**
Residential location (ref urban)	**–**	**-**	**1.89 [1.83–1.96]**

PR: prevalence risk; 95% CI: 95% confidence interval. Boldface indicates statistical significance.

## Discussion

Our findings demonstrated a consistent gradient in the dentate status, dental care utilisation and toothbrushing frequency based on educational attainment in Indonesia. People with lower educational attainment showed a higher risk of having edentulism, lower access to dental services, and poorer toothbrushing frequency.

Edentulism was related to lower levels of educational attainment. Our findings correspond quite well with the findings of a previous Indonesian study on edentulism.^17^ Higher edentulism among people with lower education compared to their counterparts was also found in many other parts of the world.^5,7,8^ Lower education and lower SES may affect dentate status by various means, including lower health literacy and lower adoption of healthier lifestyle behaviours. It may also be due to a lower ability to access dental care. Both of these outcomes were also assessed in the current study.

Lower dental service utilisation was shown among people with lower education. These results confirm a previous dental service utilisation study in Indonesia.^20^ Previous research showed inequality in dental service utilisation due to educational attainment.^9,11,20^ This finding is made more urgent by the prior finding that edentulism was also higher among those with lower educational attainment. Such inequities result from the lack of accessibility due to financial constraints. This finding was similar to a study conducted in Australia.^4^ Another study also showed that dental utilisation was related to the ability to pay.^15^ A lower educational level is likely to influence the later career path. A recent review suggested that the effect of education on individual earnings is obviously positive and large, relative to returns on other investments.^10^

In terms of the effect of education on toothbrushing frequency, a stark educational gradient is evident, i.e. people with lower education have a higher risk of poorer toothbrushing frequency. Social inequality in toothbrushing has been found by numerous other authors.^12,14,16^ One study conducted in Denmark showed that this inequality can increase over time.^12^

Results of this study confirm that education, as an important indicator of SES, was consistently related to dental condition and dental care, as inequalities by education tended to be high across all outcomes in this context. In low- and middle-income countries such as Indonesia, education reflects early-life SES.

The extent of educational inequalities in dental behaviours in Indonesia was much larger for toothbrushing behaviours than for dental care utilisation. Caries and periodontal disease are considered behavioural diseases, because they can be prevented simply by maintaining good oral hygiene and restricting the frequency of sugar consumption.^13^ Toothbrushing with fluoridated toothpaste has been recommended by the WHO to maintain good oral hygiene. Considering the great influence of education on toothbrushing frequency, there is an urgent need to create policy to tackle the problem. Although this study is cross-sectional in nature, it stands to reason that those with worse toothbrushing habits and less frequent dental service utilisation would have a higher risk of edentulism. Thus, this population is at significant disadvantage with regard to their oral health and, in turn, their oral health-related quality of life. The intervention points for health policy should urgently prioritise public health interventions to promote people’s oral health literacy, particularly among those with lower educational attainment. Chairside dental care alone will not be sufficient to address the type and levels of existing oral health inequalities.

This study is one of the few to explore the effect of socioeconomic status on teeth and dental care in a developing country. It is also the first to be conducted among an Indonesian population using a large, nationally representative sample. However, the cross-sectional study design was a limitation, as it did not allow any interpretations of causality. Some potential bias could also arise due to self-reported data and residual confounding. Education has only a few variables that can confound it, as schooling often occurs early in life. Hence, many of the other related variables, such as income, are more likely to be mediators that occur later.

Monitoring socioeconomic inequality in oral health is important in formulating appropriate public health policies. Such policies should incorporate the phenomena described above to be able to direct public health messages to the most appropriate target.

## Conclusion

The current analysis revealed socioeconomic inequality in oral health status and behaviour among Indonesians. Public health interventions should be targeted to tackle this problem by improving overall educational attainment and providing more appropriate prevention strategies and dental care among those with lower educational attainment.
